# Joint DNA-RNA-based NGS for diagnosis and treatment of a rare *CD47-MET* fusion lung adenocarcinoma which was immunoresistant and savoltinib-sensitive: a case report

**DOI:** 10.3389/fimmu.2024.1386561

**Published:** 2024-06-18

**Authors:** Rulan Wang, Yanyang Liu, Xuejiao Yu, Weiya Wang, Jiewei Liu

**Affiliations:** ^1^ Department of Medical Oncology, Cancer Center, West China Hospital, Sichuan University, Chengdu, Sichuan, China; ^2^ Lung Cancer Center, West China Hospital, Sichuan University, Chengdu, Sichuan, China; ^3^ Department of Pathology, West China Hospital, Sichuan University, Chengdu, Sichuan, China

**Keywords:** non-small cell lung cancer, *CD47-MET* fusion, *MET* p D1228H, immune microenvironment, savolitinib, case report

## Abstract

Targeted therapy and immunotherapy are both important in the treatment of non-small-cell lung cancer (NSCLC). Accurate diagnose and precise treatment are key in achieving long survival of patients. *MET* fusion is a rare oncogenic factor, whose optimal detection and treatment are not well established. Here, we report on a 32-year-old female lung adenocarcinoma patient with positive PD-L1 and negative driver gene detected by DNA-based next-generation sequencing (NGS). A radical resection of the primary lesion after chemotherapy combined with PD-1 checkpoint inhibitor administration indicated primary immuno-resistance according to her pathological response and rapid relapse. A rare *CD47-MET* was detected by RNA-based NGS, which was confirmed by fluorescence *in situ* hybridization. Multiplex immunofluorescence revealed a PD-L1 related heterogeneous immunosuppressive microenvironment with little distribution of CD4+ T cells and CD8+ T cells. Savolitinib therapy resulted in a progression-free survival (PFS) of >12 months, until a new secondary resistance mutation in *MET* p.D1228H was detected by re-biopsy and joint DNA-RNA-based NGS after disease progression. In this case, *CD47-MET* fusion NSCLC was primarily resistant to immunotherapy, sensitive to savolitinib, and developed secondary *MET* p.D1228H mutation after targeted treatment. DNA-RNA-based NGS is useful in the detection of such molecular events and tracking of secondary mutations in drug resistance. To this end, DNA-RNA-based NGS may be of better value in guiding precise diagnosis and individualized treatment in this patient population.

## 1 Introduction

Primary lung cancer remains the leading cause of cancer-related deaths worldwide. According to the National Comprehensive Cancer Network (NCCN) (https://www.nccn.org/guidelines) and The Chinese Society of Clinical Oncology (http://www.csco.org.cn/cn/) guidelines, for locally advanced or metastatic non-small cell lung cancer at initial diagnosis with clinical stage III-IV, the principle of treatment is to carry out multidisciplinary (MDT) discussion on the basis of medical treatment. With the continuous advancement of precise molecular diagnostic technology, the treatment of lung cancer has gradually evolved into individualized therapeutic strategies based on molecular and biological characteristics. It has been determined that patients with non-small cell lung cancer (NSCLC) can be divided into to two groups with distinct biological profiles based on the presence or absence of driver gene mutations. Patients with driver gene positivity, such as *EGFR* mutation, *ALK*, and *ROS*-1 fusion are more likely to benefit from targeted therapy. In contrast, patients who are negative for driver genes, especially those who are positive for PD-L1 expression, are more likely to have a survival benefit from chemotherapy and/or immunotherapy. Therefore, there is a broad consensus that patients with NSCLC should undergo the necessary genetic testing before initial treatment (1).

*MET* is a major driver gene of NSCLC. *MET* gene abnormalities include *MET* 14 exon skipping mutations, *MET* gene amplification and *MET* gene fusion (2). Of these variant forms, *MET* fusion is a rare oncogenic factor, detected in only 0.5% of patients with NSCLC (3). Since lacking optimal detection methods, it is easy to be missed and overlooked. The efficacious of *MET* fusion NSCLC to tyrosine kinase inhibitor (TKI)-targeted therapies and immunotherapy is not well known. Therefore, research into improving the understanding of the issues related to NSCLC with *MET* fusion, such as highly sensitive and specific detection methods for *MET* fusion mutations; the immune feature of tumor microenvironment; and the response to chemotherapy, targeted therapy, and immunotherapy is cardinal.

## 2 Case presentation

**Figure 1:**
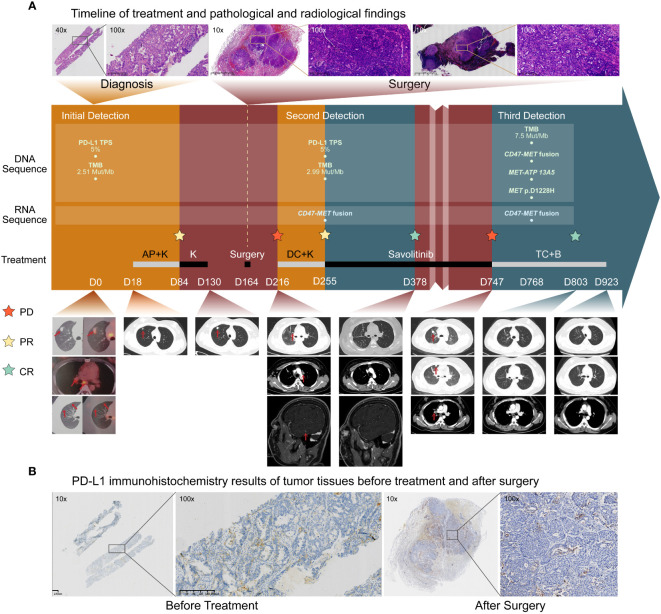
Treatment history and initial biological findings of the patient. **(A)**. Timeline of treatment and radiological findings. Hematoxylin-eosin(HE) staining before treatment showed large tumor cells. The patient received the pemetrexed and cisplatin plus Keytruda (AP+K) regimen, and tumor shrinkage and partial response was observed. Thereafter, progression occurred within a short time during immune-maintenance therapy, and the tumor recurred and metastasized soon after surgical treatment. HE staining of the primary tumor foci and the 7th station of mediastinal lymph node metastases after surgery showed many tumor cells without immunotherapy-related pathological reactions. After one cycle of the second-line docetaxel and cisplatin plus Keytruda (DC+K) regimen, postoperative RNA-based NGS of tumor tissue showed *CD47-MET* fusion. The patient received savolitinib treatment and complete imaging remission was achieved. Unfortunately, in October 2023, the patient experienced recurrence and progression. Subsequent biopsy of the lung metastases revealed adenocarcinoma characterized by *CD47-MET* fusions, *MET*-ATP13A5 fusions, and a *MET* p.D1228H mutation. Consequently, the patient was initiated on a third-line therapy comprising a combination of albumin-bound paclitaxel, carboplatin, and bevacizumab (TC+B). The red arrows show the tumor regions. **(B)** PD-L1 immunohistochemistry (IHC) results of tumor tissues before treatment and after surgery. PD- L1 expression was both positive before and after treatment, and the PD-L1 [22C3] TPS in both cases was approximately 5%.

A 32-year-old female (nonsmoker) without any symptoms was diagnosed with adenocarcinoma of the right lung with multiple mediastinal lymph nodes and suspected bilateral lung metastases (stage T1bN2M1a, IVA) (**Figure 1A**) according to the PET/CT (positron emission tomography/computedtomography) at the local hospital of her first visit. Physical examination showed no abnormalities. DNA-based next-generation sequencing (NGS) (BGI Genomics company of Shandong China, MGISEQ-2000 platform, panel of 688 genes) (**Supplementary 1 Methods A1**) showed no driver gene mutations with tumor mutation burden as 2.51 Muts/Mb, while PD-L1 TPS was 5% (**Supplementary 2 Table A1**) (**Figure 1B**). Following four cycles of chemotherapy combined with pembrolizumab immunotherapy, a partial response was achieved. However, the lesion exhibited slow growth during the immunotherapy maintenance phase (**Figure 1A**). When the patient visited our hospital for further treatment advice, we carefully analyzed the patient’s PET/CT images and found that the patient’s bilateral lung nodules in the initial PET/CT were small and not accompanied by elevated metabolic abnormalities. Therefore, we thought that the clinical diagnosis of stage IVA had not been fully established in this patient. As the patients’ primary foci slowly increased in size with no clear signs of progression for mediastinal lymph nodes, and the small nodules in both lungs did not show definitively, a multidisciplinary treatment team including surgeons indicated the limited value of internal medicine and potential benefit of surgery. Also, there was no absolute contraindication for surgery, and the young patient had a strong desire for surgery. Thus, the patient underwent radical resection of the upper lobe of the right lung and systemic lymph node dissection. Intraoperatively, a widely disseminated pleural nodule in the right pleura was found, and maximal resection, cauterization, and pleural fixation of the pleural nodule were performed. Postoperative pathology suggested hypodifferentiated adenocarcinoma with residual cancer cells (~95%) with no necrotic tissue or immunotherapy-related pathological changes. Tumor metastasis extended across the visceral pleura and multiple mediastinal lymph nodes, lacking discernible treatment responses. DNA-based NGS (Burning Rock Biotech company of Chengdu China, OncoScreen PlusⓇ, panel of 520 genes) was repeated with no new findings of driver genes (**Supplementary 2 Table A1**). Tumor mutation burden was 2.99 Muts/Mb while PD-L1 TPS was 5% in postoperative tumor tissues (**Figure 1B**). Unfortunately, new nodes in the right temporal lobe, intrapulmonary and mediastinal, showed suspicious metastases 1 month after surgery (**Figure 1A**).

**Figure 2:**
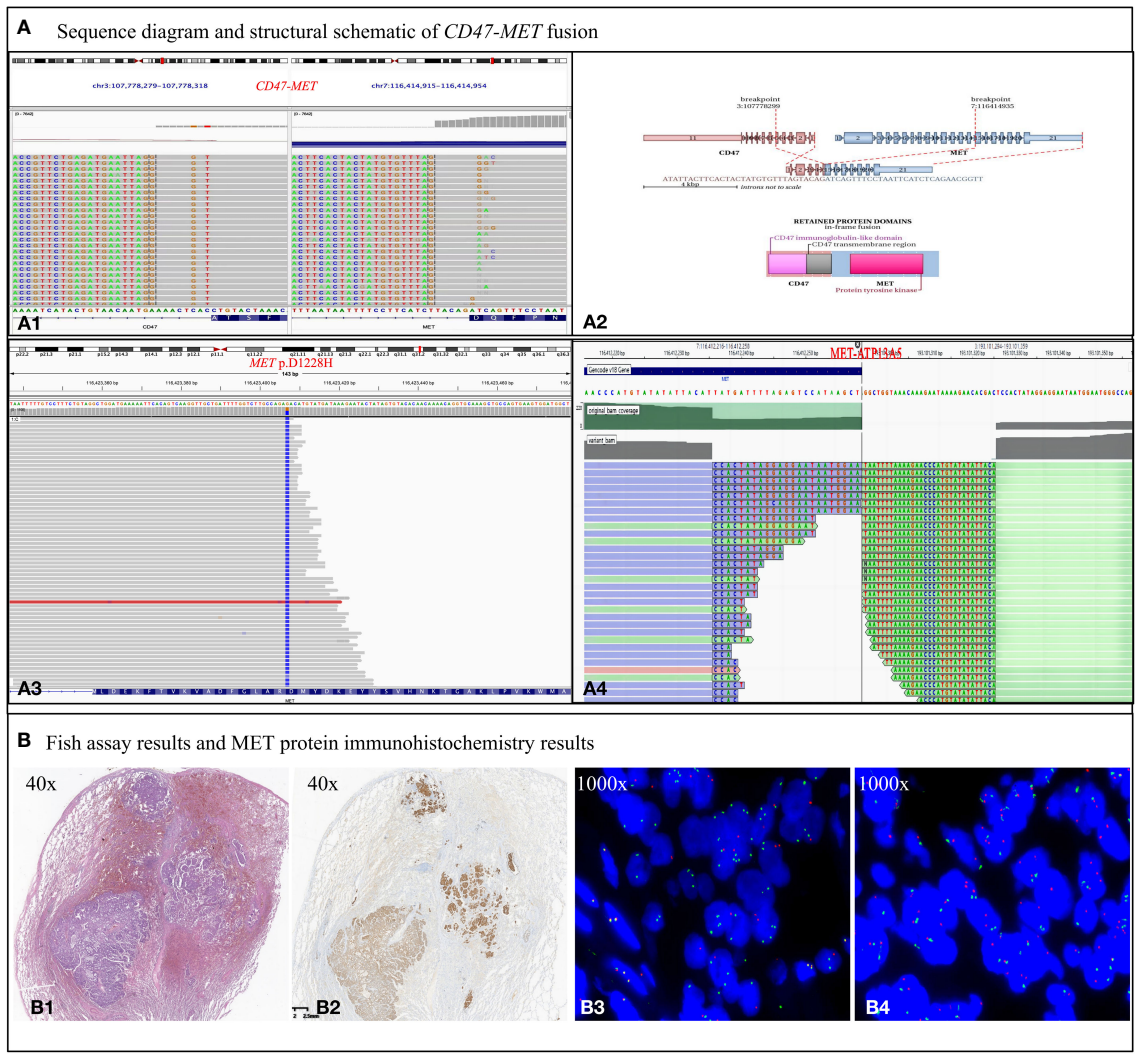
Biological examination of *MET* fusion. **(A)** Sequence diagram and structural schematic of *MET* fusion and *MET* p.D1228H mutation. Exon 5 of the CD47 gene and exon 15 of the *MET* gene were fused. The fusion gene retains the 5’ end of CD47 and the 3’ end of *MET* gene, which are thought to form a functional fusion protein at the RNA level. The fusion gene contains the regulatory dimerization domain (RDD) of the CD47 gene and the intact TKD of the *MET* gene. RDD drives the constitutive activation of the TKD, which theoretically produces kinase proteins that activate the downstream signaling pathway of *MET* **(A1, 2)**. However, the *MET* p.D1228H mutation, which is in the kinase domain of the *MET* gene, confers resistance to TKIs **(A3)**. Nevertheless, the fusion event between ATP13A5 and *MET* occurs within the promoter region and is not anticipated to result in the formation of a novel fusion protein **(4A)**. **(B)** Results of MET protein IHC assay and FISH assay. **(B1)** Tumor showing invasive growth and composed of solid (~ 70%), cribriform (20%), and acinar (10%) architecture, with a scattered signet ring cell carcinoma component. **(B2)** Met protein IHC assay showing c-Met protein expression exhibiting a moderate to strong positive staining pattern in the cell membrane and is homogeneous among morphological variants. **(B3)** *MET* break-apart signals in 65% of tumor cells, demonstrating C-*MET* gene translocation. **(B4)** No heterogenous break-apart percentage in different areas of the histological variants. A normal *MET*/CEP7 ratio (1.6) and no copy number gain of *MET* gene (2.4 copies per cell) is present based on counting 50 tumor cells within the tumor area, suggesting no C-*MET* gene amplification.

To identify any potential driver gene mutations, we performed RNA-based NGS (Burning Rock Biotech company of Chengdu China, OncoRNA assay) on the postoperative specimen with detection of a rare mutation of *CD47-MET* fusion (**Figures 2A1, A2**). The fusion retained the MET’s tyrosine kinase structural domains (TKD) and consistently expressed c-Met protein upon activation of the regulatory dimerization domain formed by CD47 (**Figures 2B1, B2**). Fluorescence in the situ hybridization (FISH) assay validated the C-*MET* translocation (**Figure 2B3**) and no copy number gain of *MET*, suggesting no C-*MET* amplification (**Figure 2B4**). Because the benefits of chemotherapy and immunotherapy are limited, savolitinib, a new *MET*-TKI, was prescribed. The lung and head lesions shrank and disappeared in 4 months (**Figure 1A**). Within 7 months, blood carcinoembryonic antigen (CEA) decreased to become normal, achieving complete remission (**Supplementary 3 Figure A1**).

**Figure 3:**
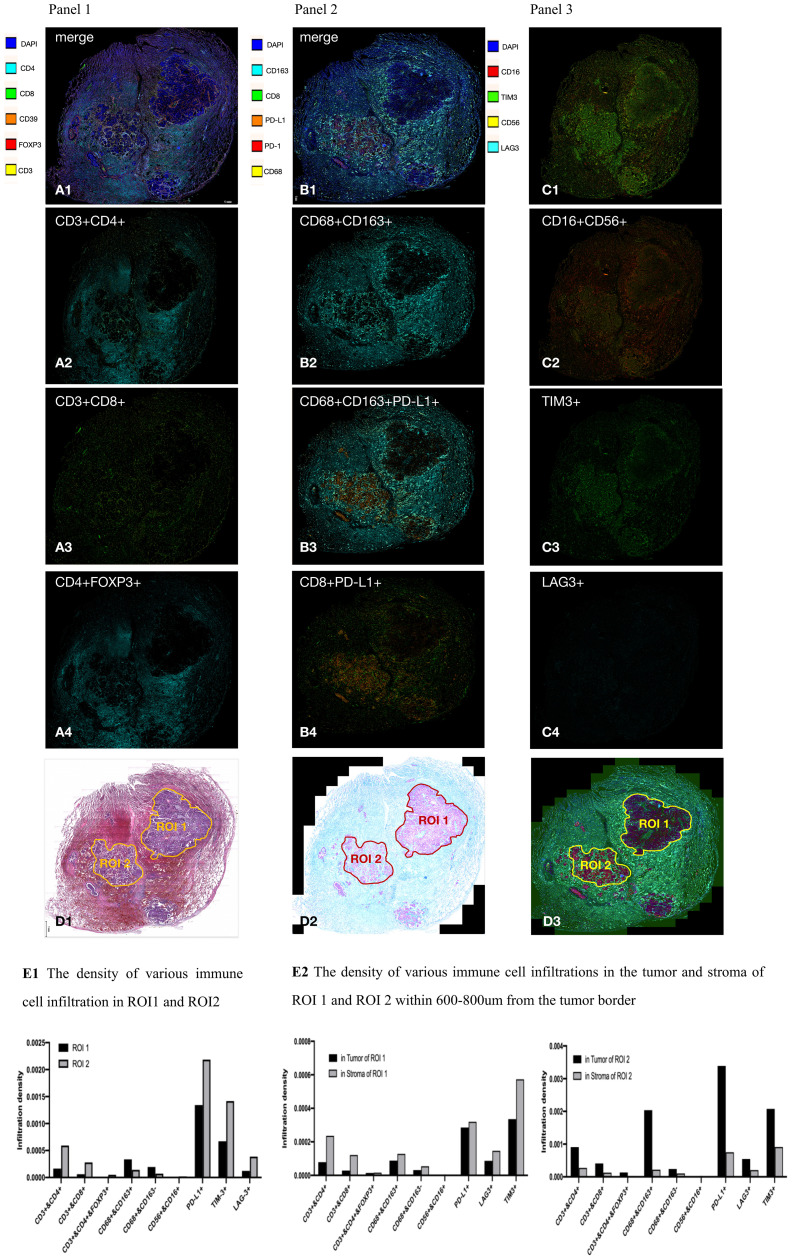
Results of multiplex immunofluorescence detection of tumor microenvironment. **(A–C)** In general, CD4+ T cells (CD3+CD4+), CD8+ T cells (CD3+CD8+), and M2-type tumor-associated macrophage (TAM) cells (CD68+CD163+); Treg cells (CD3+CD4+FOXP3+); and NK cells (CD16+CD56+) had a significantly lower percentage of infiltration in the tumor area than in the tumor stroma. CD4+ T cells, CD8+ T cells, M2 type TAM cells, PD-L1, and TIM3 had heterogeneous distribution in different tumor areas; **(D)**, respectively, in HE images, mIF bright field, and mIF fluorescence maps delineating ROI 1 and ROI 2; **(E1)**, the infiltration density of CD4+ T cells, CD8+ T cells, and Treg cells within ROI 2 was significantly higher than that in ROI 1. Expression of PD-L1 and TIM3 within ROI 2 was significantly higher than that in ROI 1; **(E2)** CD4+ T cells, CD8+ T cells, Treg cells, and M2 TAM infiltrated the peripheral stroma region more significantly than the tumor region in ROI 1, but the distribution was reversed in ROI 2.

To explore the possible connection between *CD47-MET* fusion and immunogenic resistance, we performed mIF (multiple immunofluorescence) assays on the surgical resection specimens. Compared to paraneoplastic tissue, the infiltration ratio of CD4+ T, CD8+ T, and M2-type tumor-associated macrophages (TAM), T-regulatory (Treg), and NK cells was relatively low in tumor tissue, whereas the infiltration of immune cells in different tumor regions presented with significant heterogeneity (**Figures 3A–C**). We identified two tumor regions with different degrees of immune cell infiltration in the mIF images—region of interest (ROI) 1 and ROI 2 (**Figure 3D**), and then we compared various immune cells’ infiltration densities in the two regions. Infiltration densities of CD4+ T, CD8+ T, and Treg cells, excluding M2-TAMs, within ROI 2 were considerably higher than those of ROI 1, as were the positive expression rates of PD-L1 and TIM3 (**Figure 3E1**; **Supplementary 4 Tables B1–B6**). To further explore the immune microenvironment of the tumor, we analyzed the densities of various immune cells within and outside the boundaries of ROIs 1 and 2 at distances of 0–200, 201–400, 401–800, and 801–1000 µm. The infiltration degree of CD4+ T, CD8+ T, and Treg cells, and M2-TAMs were substantially higher in the peripheral stroma than in the tumor area in ROI 1. However, these cells were significantly higher in the tumor area than in the peripheral stroma in ROI 2 (**Figure 3E2**; **Supplementary 4 Tables B7–B9**).

As of September 2023, a progression-free survival of >12 months was achieved. Unfortunately, the patient was found to have significantly elevated CEA in October 2023, and chest CT suggested recurrence of the operative stump with new metastases in the mediastinal lymph nodes, both intrapulmonary, right pleura, and chest wall (**Figure 1A**). A repeat puncture biopsy of lung metastases showed adenocarcinoma, while DNA NGS (GenePlus company of Chengdu China, Gene+Seq2000, panel of 1021 genes) combined with RNA NGS (GenePlus company of Chengdu China, tumor-related RNA gene test platform) suggested *CD47-MET* fusion with *MET* p.D1228H mutation (55.1%) (**Figure 2A3**). Additionally, the RNA NGS analysis revealed a novel *MET-ATP*13A5 fusion event that was not predicted to result in a functional protein product (**Figure 2A4**). The patient stopped oral savolitinib and was initiated on third-line treatment of albumin paclitaxel, carboplatin, and bevacizumab. Following two treatment cycles, the lymph node and bilateral lung metastases exhibited complete regression, accompanied by a normalization of CEA levels (**Figure 1A**). To date, the patient has finished the 6 cycles of third-line therapy without any tumor-related symptoms and CEA remains at normal levels. The continuing follow-up is currently undergoing.

## 3 Discussion

*CD47-MET* fusions are extremely rare according to global reports. To the best of our knowledge, this is the first case to report the immune microenvironment status of a patient with *CD47-MET* fusion and the secondary resistance mutation site after targeted therapy. The clinical diagnosis and treatment process also presents new insights.

First, the results of the patient’s multiple genetic tests suggested the necessity of joint DNA-RNA NGS in this patient population. Currently, there have been several papers highlighting the importance of evaluating gene fusions on RNA testing to avoid false negatives, and clinicians are conscious to assess the biomarkers before starting immunotherapeutic treatment (4, 5). DNA NGS exhibits good sensitivity for detecting point mutations, small insertions/deletions, and large chromosome copy number variations. The shortcomings of DNA-NGS of fusion genes are as follows: the probes cannot fully cover the introns, which may lead to missed detection; due to many repeating sequences of the introns, it is difficult to design the probes, generate raw signal algorithms, and compare sequencing data; complex structural variants located near the cut site are difficult to detect directly, and can only to be speculated (6). Additionally, gene fusions formed by RNA splicing, which is a process whereby fusion RNAs can be formed by splicing between exons from two adjacent or non-adjacent genes during transcription, are currently unable to be detected by DNA-NGS (7). Contrarily, the design of RNA-NGS probes eliminates introns, and directly detects exon deletions at the transcriptional level, which is simpler, more direct, and more functionally relevant (2). Previous publications have shown that RNA-based NGS in cases with negative DNA-NGS findings resulted in the detection of gene fusions/rearrangements or *MET* exon14 skipping in 10–14% of samples (5). Therefore, the NCCN guideline suggests that, for patients who in broad panel testing do not have identifiable driver oncogenes, consider RNA-based NGS if not already performed, to maximize detection of fusion events (1). Moreover, rare fusion partners, exon breakpoint fusions, and intergenic fusions detected at the DNA level can be further validated at the RNA or protein level, especially in young, female, nonsmoking, mucinous/solid lung adenocarcinoma patients with low TMB. Additionally, we found the FISH assay can be considered for clinical use to compensate for conventional DNA testing in our case. If *MET* mutations are not detected by DNA-based NGS, but initial screening of Met IHC yields positive results, incorporating results could suggest for subsequent RNA-based NGS.

Second, this is the first report on *CD47-MET* fusion NSCLC with PD-L1 positive expression with primary resistance to anti-PD1 immunotherapy. During maintenance treatment with PD-1 blockers, *CD47-MET* fusion NSCLC progressed rapidly. The surgically resected specimen showed a large proportion of residual cancer cells with a low percentage of fibrotic and inflammatory tissue, while few immune effector T and NK cells were detected at the tumor site by mIF (**Figures 3A–C**), indicating the state of immunotherapy resistance. The reason for low immune cell infiltration in tumor tissues of *CD47-MET* fusion NSCLC is currently unknown. According to existing reports, activation of the *MET* signaling pathway can induce the state of an immunosuppressive microenvironment through diverse mechanisms, including reducing the infiltration of CD8+ cytotoxic T cells and NK cells (8), down-regulating the immune stimulatory factors CD137, CD252, and CD70 (9), inducing a high infiltration of Tregs in the tumor microenvironment (10, 11), and transforming M1 type TAM (immune activation type) into M2 type TAM (immunosuppressive type) (12, 13), thereby engendering resistance to immunotherapy. Herein, PD-L1 showed heterogeneous expression in different tumor regions in this case, and the variation in its expression intensity was consistent with the infiltration levels of CD4+ T cells, CD8+ T cells, and Tregs. However, despite the significant differences in the proportion of immune cells in different ROIs, none had a cytotoxic effect on tumor cells. This finding suggests that in *CD47-MET* fusion NSCLC, PD-L1 expression does not predict the efficacy of PD-1 inhibitors, which may be related to an immunosuppressive microenvironment.

Third, this case is the first to report a resistance mechanism following a *CD47-MET* fusion mutation as *MET* p.D1228H in NSCLC after savoltinib treatment. According to literature, the *MET* p.D1228 mutation is located in the kinase structural domain of the *MET* gene protein, and p.D1228 is one of the common resistance mutation sites in patients with *MET* exon14 skipping or *MET* amplification following treatment with a type I *MET* TKI (14, 15). In addition, Prof. Jing-Ji Yang’s team reported a case of *MET* D1228N mutation in a patient with *EPHB4-MET* fusion on crizotinib and verified that the mutant tumor may be sensitive to tivantinib, a type III *MET* inhibitor, in a PDX mouse model (16). Another case with *MET* exon14 skipping developed the p.D1228N mutation after being treated with crizotinib, which was resistant to cabozantinib and progressed 4 months after achieving partial response (17). Our case, as well as the aforementioned reports, provides a theoretical basis for backline drug development by providing a possible secondary resistance mechanism of *MET* fusion genes after treatment with *MET* TKI drugs.

## 4 Conclusion

We report for the first time that *CD47-MET* fusion NSCLC is primarily resistant to immunotherapy, which may be associated with *MET* activation-induced immunosuppressive microenvironments while positive PD-L1 expression is not a predictor of immunotherapeutic efficacy in this type of NSCLC. For patients who are more prone to having positive driver genes based on their clinical profiles, especially those with primary resistance to immunotherapy, joint DNA-RNA-based NGS may be of better value in guiding precise molecular diagnosis and individualized treatment.

## Data availability statement

The original contributions presented in the study are included in the article/**Supplementary Material**. Further inquiries can be directed to the corresponding authors.

## Ethics statement

The studies involving humans were approved by the ethics committees of West China Hospital of Sichuan University. The studies were conducted in accordance with the local legislation and institutional requirements. The human samples in this study were obtained from lung needle biopsies and surgical resections. Written informed consent for participation was not required from the participants or the participants’ legal guardians/next of kin in accordance with the national legislation and institutional requirements. Written informed consent was obtained from the individual(s) for the publication of any potentially identifiable images or data included in this article.

## Author contributions

RW: Data curation, Formal analysis, Investigation, Visualization, Writing – original draft, Writing – review & editing. YL: Data curation, Formal analysis, Funding acquisition, Writing – original draft, Writing – review & editing. XY: Data curation, Visualization, Writing – review & editing. WW: Conceptualization, Data curation, Formal analysis, Funding acquisition, Supervision, Writing – original draft, Writing – review & editing. JL: Conceptualization, Data curation, Formal analysis, Funding acquisition, Resources, Supervision, Validation, Writing – original draft, Writing – review & editing.

## Supplementary material

Supplementary 1 Methods A1 Materials and methods used in the present study.

Supplementary 2 Table A1 Comparison of the patient’s genetic test results.

Supplementary 3 Figure A1 Carcinoembryonic antigen (CEA) levels during the course of disease.

Supplementary 4 (xls). Table B1, Infiltration of various immune cells of Panel 1; Table B2, Infiltration of various immune cells of Panel 1 in ROI 1 and ROI 2; Table B3, Infiltration of various immune cells of Panel 2; Table B4, Infiltration of various immune cells of Panel 2 in ROI 1 and ROI 2; Table B5, Infiltration of various immune cells of Panel 3; Table B6, Infiltration of various immune cells of Panel 3 in ROI 1 and ROI 2; Table B7 Densities of various immune cells within and outside the boundaries of ROI 1 and ROI 2 at different distances (Panel 1); Table B8 Densities of various immune cells within and outside the boundaries of ROI 1 and ROI 2 at different distances (Panel 2); Table B9 Densities of various immune cells within and outside the boundaries of ROI 1 and ROI 2 at different distances (Panel 3).
