# Supplementary material for: Joint DNA-RNA-based NGS for diagnosis and treatment of a rare CD47-MET fusion lung adenocarcinoma which was immunoresistant and savoltinib-sensitive: a case report
Source: Front Immunol. 2024 Jun 18;15:1386561. doi: 10.3389/fimmu.2024.1386561 (PMC11217332; doi:10.3389/fimmu.2024.1386561)
Supplement: Supplementary file 1 [file DataSheet_1.pdf]

CARE Checklist of information to include when writing a case report

(cc) BY-NC-ND

Topic	Item No	Checklist item description	Reported on Page Number/Line Number	Reported on Section/Paragraph
Title	1	The diagnosis or intervention of primary focus followed by the words "case report"	Page 1/ Line 1	Title
Key Words	2	2 to 5 key words that identify diagnoses or interventions in this case report, including "case report"	Page 2/Line 28-29	Key words
Abstract (Structured summary)	3a	Background: state what is known and unknown; why the case report is unique and what it adds to existing literature.	Page 3/Line 31-34	Abstract
	3b	Case Description: describe the patient's demographic details, main symptoms, history, important clinical findings, the main diagnosis, interventions, outcomes and follow-ups.	Page 3/Line 35-45	Abstract
	3c	Conclusions: summarize the main take-away lesson, clinical impact and potential implications.	Page 3/Line 46-51	Abstract
Introduction	4	One or two paragraphs summarizing why this case is unique **(may include references)**	Page 4/Line 54-80	Introduction/Para 1-2
Patient Information	5a	De-identified patient specific information	Page 4/Line 82	Case presentation/Para 1
	5b	Primary concerns and symptoms of the patient	Page 4/Line 82-86	Case presentation/Para 1
	5c	Medical, family, and psycho-social history including relevant genetic information	Page 4/Line 85-89	Case presentation/Para 1
	5d	Relevant past interventions with outcomes	Page 4/Line 90-92	Case presentation/Para 1
Clinical Findings	6	Describe significant physical examination (PE) and important clinical findings	Page 4/Line 86	Case presentation/Para 1
Timeline	7	Historical and current information from this episode of care organized as a timeline	Page 12/Fig 1	Figure/Fig 1
Diagnostic Assessment	8a	Diagnostic testing (such as PE, laboratory testing, imaging, surveys).	Page 4/Line 85-86	Case presentation/Para 1
	8b	Diagnostic challenges (such as access to testing, financial, or cultural)	Page 4-5/Line 93-103	Case presentation/Para 1
	8c	Diagnosis (including other diagnoses considered)	Page 4-5/Line 82-100	Case presentation/Para 1
	8d	Prognosis (such as staging in oncology) where applicable	Page 6/Line 159-163	Case presentation/Para 1
Therapeutic Intervention	9a	Types of therapeutic intervention (such as pharmacologic, surgical, preventive, self-care)	Page 5,6/Line 103, 124,,158	Case presenta/Para 1-4
	9b	Administration of therapeutic intervention (such as dosage, strength, duration)	Page 5,6/Line 103, 126,161	Case presenta/Para 1-4
	9c	Changes in therapeutic intervention (with rationale)	Page 5,6/Line 114, 149	Case presenta/Para 1, 4

Follow-up and Outcomes	10a	Clinician and patient-assessed outcomes (if available)	Page 6/Line 159-162	Case presentation/Para 4
	10b	Important follow-up diagnostic and other test results	Page 6/Line 162	Case presentation/Para 4
	10c	Intervention adherence and tolerability (How was this assessed?)	Page 6/Line 161-162	Case presentation/Para 4
	10d	Adverse and unanticipated events	Page 6/Line 161-162	Case presentation/Para 4
Discussion	11a	A scientific discussion of the strengths AND limitations associated with this case report	Page 6-7/Line 170-198	Discussion/ Para 2
	11b	Discussion of the relevant medical literature **with references**	Page 6-8/Line 170-234	Discussion/ Para 2-4
	11c	The scientific rationale for any conclusions (including assessment of possible causes)	Page 6-8/Line 170-234	Discussion/ Para 2-4
	11d	The primary “take-away” lessons of this case report (without references) in a one paragraph conclusion	Page 8/Line 236-242	Conclusion/ Para 1
Patient Perspective	12	The patient should share their perspective in one to two paragraphs on the treatment(s) they received	N/A	N/A
Informed Consent	13	Did the patient give informed consent? Please provide if requested	**Yes** ☑	**No** ☐

Article Information: http://dx.doi.org/10.21037/tcr-22-141
*As the checklist was provided upon initial submission, the page number/line number reported may be changed due to copyediting and may not be referable in the published version. In this case, the section/paragraph may be used as an alternative reference.
