# Supplementary material for: Joint DNA-RNA-based NGS for diagnosis and treatment of a rare CD47-MET fusion lung adenocarcinoma which was immunoresistant and savoltinib-sensitive: a case report
Source: Front Immunol. 2024 Jun 18;15:1386561. doi: 10.3389/fimmu.2024.1386561 (PMC11217332; doi:10.3389/fimmu.2024.1386561)
Supplement: Supplementary 2 Table A1 — Comparison of the patient’s genetic test results. [file Table_1.docx]

**Table 1** Comparison of patient's genetic test results pre-treatment and post-treatment.

| **Detection type** | **DNA sequence** | | | | | | | | | | | | **RNA**  **sequence** |
| --- | --- | --- | --- | --- | --- | --- | --- | --- | --- | --- | --- | --- | --- |
| **Detection items** | ***TP53*** | | | ***CDK6*** | ***NYAP2*** | ***NTRK1*** | | **TMB**  ( Muts/Mb) | **PD-L1**  **TPS** | ***MET*** | | | **/** |
| **Mutation Locations** | p.R249K | p.H214Y | P.T125A | Copy number increasing | Exon 4 p.T382M | Exon 8 p.P351R | Exon 9 | **/** | **/** | **/** | | | **/** |
| **Initial Detection** | 55.35% | **/** | **/** | 4 | 18.18% | 12.22% | **/** | 2.51 | 5% | / | | | Untested |
| **Second**  **Detection** | 27.26% | 2.36% | / | **/** |  | **/** | 9.44% | 2.99 | 5% | / | | | *CD47-MET* fusion |
| **Third**  **Detection** | 75.6% | / | 38.2 | 1.8 |  | **/** | / | 7.5 | / | *CD47-MET* fusion | *MET-ATP13A5* | *MET* p.D1228H | *CD47-MET* fusion |
